# Home and Health in the Third Age — Methodological Background and Descriptive Findings

**DOI:** 10.3390/ijerph110707060

**Published:** 2014-07-11

**Authors:** Maya Kylén, Henrik Ekström, Maria Haak, Sölve Elmståhl, Susanne Iwarsson

**Affiliations:** 1 Department of Health Sciences, Lund University, SE-22100, Sweden; E-Mails: Henrik.ekstrom@med.lu.se (H.E.); Maria.haak@med.lu.se (M.H.); Solve.elmstahl@med.lu.se (S.E.); Susanne.iwarsson@med.lu.se (S.I.); 2 Skåne University Hospital, SE-205 02, Sweden

**Keywords:** activities of daily living, aging, housing, P-E fit, younger old

## Abstract

*Background:* The understanding of the complex relationship between the home environment, well-being and daily functioning in the third age is currently weak. The aim of this paper is to present the methodological background of the Home and Health in the Third Age Study, and describe a sample of men and women in relation to their home and health situation. *Methods and Design:* The study sample included 371 people aged 67–70, living in ordinary housing in the south of Sweden. Structured interviews and observations were conducted to collect data about objective and perceived aspects of home and health. *Results:* The majority of the participants were in good health and had few functional limitations. Women had more functional limitations and reported more symptoms than men. Environmental barriers were found in every home investigated; the most were found in the kitchen and hygiene area. Environmental barriers were more common in multi-family than in one-family dwellings. *Discussion:* This study will increase our knowledge on home and health dynamics among people in the third age. The results have potential to contribute to societal planning related to housing provision, home care and social services for senior citizens.

## 1. Introduction

It is widely known that the world’s age composition is radically changing towards a higher proportion of older people than ever seen before [1]. For example, in Sweden 19.1% of the population are 65 years old or older, and this proportion is expected to increase to 25% by 2060 [2]. Thus, more than one out of five will be over the age of 65 by the year 2030. Since 1990, the life expectancy after retirement age has risen for 65-year-olds as well as for those who have reached the age of 80 [3].

While aging can be viewed from a chronological, biological, psychological or social perspective, chronological and biological aging are not equivalent for all individuals. Hence, different stages of the aging process can be defined in terms of the fourth and third age [3], in terms of population or personal characteristics [4]. For the present study we used a definition based on personal characteristics, where the fourth age is characterized by frailty, cognitive decline and functional loss, and the third age by independence, social engagement and good health. Commonly, the third age is represented by individuals recently retired from work, but the fourth and third ages are dynamic in the sense that they cannot be defined by specific age ranges.

As the proportion of the population aged 65 years or older is increasing and the home environment is an important arena in order to support independence and well-being in old age [5], home and health dynamics play an increasingly important role for societal planning in terms of housing provision, health care and social services. There is a growing body of research with regard to home and health in very old age, that is, studies involving individuals who have reached the fourth age [6,7,8,9]. However, research is lacking on the complex interaction between the home environment, well-being and daily functioning among people in the third age. The health development along the process of aging is the subject of an extensive amount of studies with some controversial and contradictory results [1,10]. That is, functional limitations and symptoms do escalate with age; hearing impairments, mobility restrictions, depression, fatigue and joint pain increase with age and are common among people 77 years and older [11]. Even so, research indicates that there is a positive development in aspects of health such as independence in daily activities [3], which might be an effect of good housing standard and better technical equipment in the home. Moreover, housing adaptations and assistive devices (ADs) seem to compensate for deteriorating functional capacity as we age [3,10], but as yet few studies exist where home and health dynamics in the third age have been the focus of interest.

In Sweden, the majority of people in the third age live in the same types of ordinary housing as the younger population [10]. In the 65- to 74-year-old age group, 65% live in one-family, whereas 36% live in multi-family dwellings, and it is only among those 85 years old and older that a shifting trend towards living in assistive living facilities is seen. Overall, as a result of policy changes in Sweden and other western countries that involve a shift from institutional care to community services, a growing number of older people remain in their ordinary homes well into old age [10,12]. The results of studies involving very old people show that the home environment plays a central role in supporting autonomy, social inclusion and well-being in the aging population [13,14], but little is known regarding the situation during earlier phases of the process of aging.

While most studies on aging cover a multitude of information on the aging person, empirical aging research that takes a balanced person and environment view remains rare [15]. Theoretically, the relation of housing and health is closely linked to Lawton and Nahemow’s [16,17] ecological theory of aging. According to this theory, the interacting combination of an individual’s competence and the physical demands of the environment (person-environment fit—P-E-fit) is important for an individual’s level of functioning. Moreover, the docility hypothesis suggests that the lower the individual’s competence, the greater the impact of the environment on the individual’s ability to compensate for negative consequences. Though, it is important to note that when studying the relation between the aging person and the environment, the environment needs to be understood as a dynamic and context-bound phenomenon which encompasses a collection of objective as well as perceived meaning-related aspects such as emotions of a person in relation to his/her home [18]. As research on very old people has shown that it is not adequate to only measure objective aspects of housing such as physical environmental barriers, accessibility (an aspect of P-E fit) and housing standard, it is also necessary to account for perceived aspects of housing [9].

Already a decade ago, Gitlin [12] urgently called for a broader diversity of research regarding home environments that includes older people from different age cohorts with different levels of competencies and life experiences. For example, individuals born in the 1940s will have expectations and demands different from those of earlier generations. They work into a higher age, move more often, and have an overall active lifestyle [19]. In order to increase the knowledge on health trajectories related to housing, studies need to involve various cohorts of older people, with the potential to expose contrasts and shed new light on home and health dynamics along the process of aging. Thus, in order to complement the existing knowledge on home and health dynamics among people in the fourth age (see e.g., [20]), the aim of this paper is to present the methodological background of the Home and Health in the Third Age Study, and describe a sample of men and women aged 67 to 70 in relation to their home and health situation.

## 2. Methods

### 2.1. The SNAC/GÅS Project

The Home and Health in the Third Age Study is a part of the Gott Åldrande i Skåne (GÅS) [Good Aging in Skåne] project which is one arm of the largest on-going longitudinal population-based sequential cohort study on aging in Sweden (Swedish National Study on Aging and Care) (SNAC) [21,22], started in 2001. In Skåne County, the SNAC/GÅS database currently comprises 2931 people 60 to 93 years old. A randomised population register selection for the age groups 60, 66, 72, 78, 81, 84, 87, 90, and 93 years has been made. For the present study we were restricted to using the SNAC/GÅS subsample including the age cohort 66 years at inclusion. The participants were recruited from five municipalities that differ in sizes and cover rural as well as urban and semi-urban areas; Malmö (urban), Eslöv, Hässleholm, Osby and Ystad (rural, semi-urban and urban). The cohorts are followed up in recurring evaluations every third (the older cohorts) or sixth (the younger cohorts) year [21] with the purpose to increase the knowledge on normal aging, identify predictors for chronic diseases and functional decline, as well as describe the need and use of health care.

### 2.2. The Home and Health in the Third Age Study

Approximately two years after the ordinary SNAC/GÅS data collection, a cohort of 673 participants aged 67–70 years was selected from the SNAC/GÅS database and invited to take part in the Home and Health in the Third Age Study. The core methodology derives from instrumentation used to capture aspects of housing and health within the cross-national European project “Enabling Autonomy, Participation, and Well-Being in Old Age: The Home Environment as a Determinant for Healthy Aging” (ENABLE-AGE) [23].

### 2.3. Recruitment Process

A letter to all potential participants, comprising information about the study and asking them to participate was sent out by mail, asking them to return a letter of consent or decline. The individuals who consented to participate were contacted by a project administrator via telephone to book an appointment for data collection during a home visit. Due to the large number of participants, it was not possible to contact all within a set timeframe; it took anywhere between one week and two months until a participant was called.

### 2.4. Instruments

#### 2.4.1. Descriptive Variables

Socio-demographic descriptive variables included were age, sex, marital status, level of education, and type of housing. Age was calculated precisely by computing the difference between the date of interview and the date of birth. Marital status was dichotomized into married/cohabitant and unmarried/divorced/ widowed, with the intent to capture whether the participants lived alone or cohabitated. Level of education was divided into three categories: elementary school/less, secondary school, or one year more than secondary school/university degree. Project administrators trained for the data collection noted if the participants lived in a one or multi-family dwelling, and the participants were asked if they owned or rented their home. Type of housing was then divided into three categories: one-family house, rented or owned apartment in multi-family building.

#### 2.4.2. Objective Aspects of Health

##### Activities in Daily Life (ADL)

The ADL Staircase was used to assess dependence in activities of daily life (ADL). The instrument includes five items of personal activities of daily living (P-ADL; feeding, transfer, toileting, dressing, bathing) and four instrumental ADL items (I-ADL; cooking, transportation, shopping, cleaning). The instrument is administrated using a combination of interview and observation. The assessment is recorded on a three-point scale (independent, partly dependent and dependent), with dependence defined in terms of assistance from another person. The ADL Staircase is used to summarize an individual’s overall ADL ability where the degree of dependence is ranked from 0 (independent in all activities) to 9 (dependent in all activities). The instrument is reliable and valid for the assessment of older people’s functional ability [24].

#### 2.4.3. Perceived Aspects of Health

##### Difficulty in ADL Performance

In addition to the ADL Staircase, a question on self-perceived difficulty in ADL was used to capture the heterogeneity within the group of participants that were rated as independent. Directly after a participant had been rated as independent in an ADL Staircase item, the project administrator asked whether he/she performed the specific task with or without difficulty [25].

##### Functional Independence

Perceived functional independence (PFI) was addressed by the question “All in all, how would you evaluate your own independence, *i.e.*, in performing activities of daily living?”, scored from 0 (completely dependent) to 10 (completely independent); only the endpoints are defined.

##### Depressive Symptoms

To capture depressive symptoms, the 15 item version of the Geriatric Depression Scale (GDS) [26] was used. The project administrator presented each item and asked the participants to answer yes or no based on how they felt over the past week. Five items indicate a depressive symptom when rated negatively while the remaining 10 items indicate a depressive symptom when rated positively. Each “depressive” answer equals 1 point, with possible scores ranging from 0 to 15. Chronbach’s alpha on our dataset indicates acceptable internal consistency; α = 0.77.

##### Falls

A short version of the Falls Efficacy Scale-International (FES-I) [27] was used to assess fear of falling (FOF). The short FES-I contains seven items that exemplify different social and physical activities performed inside and outside the home (getting dressed/undressed, taking a bath/shower, getting in/out of a chair, going up/down stairs, reaching for something above your head/on the ground, walking up/down a slope, going out to a social event). The participants were asked to state their level of concern about falling when performing the given activity. In case the activity was not currently performed, the participants were asked to think about how afraid they would be if doing it. The instrument has four response alternatives ranging from 1 (not at all concerned) to 4 (very concerned of falling). The scores are added to a total score which can range from 7 (no concern about falling) to 28 (severe concern about falling) [28]. The participants were asked four additional questions regarding falls; “During the past year, how often have you fallen?” (never, once, and more than once) and if yes;” Where did you fall?”; “How did it happen?”, and, “Did you hurt yourself so that you needed medical care?”

##### Symptoms

A checklist consisting of 30 items was used to dichotomously (yes/no) assess the number of symptoms in seven different domains (depression, tension, gastrointestinal, musculoskeletal, metabolism, heart lung, head symptoms). The participants were asked to answer yes when they had experienced a symptom during the past three months [29]. In addition to the 30 items, three items (frequency in passing urine, incontinence, dental problems) introduced by the ENABLE-AGE consortium were included.

##### Perceived Health and Mobility

The question “In general would you say your health is…?” from the SF-36 questionnaire [30] was used to capture a global self-rating of perceived health. The scale has five response alternatives ranging from 1 (excellent) to 5 (poor). Using the same response alternatives, the participants were also asked, “How would you rate your physical mobility at the moment?”

##### Life Satisfaction

Life satisfaction was assessed through a single study-specific question, “On the whole, what do you think about your life right now?” A five-point rating scale ranging from 1 (very good) to 5 (very bad) was presented to the participants.

##### Psychological Wellbeing

The Ryff scales of Psychological Wellbeing (PWQ) [31] incorporate several different theoretical perspectives and measure positive psychological functioning. A short form with 19 items divided in two domains, autonomy (10 items) and purpose in life (nine items), was used. Statements were presented to the participants with the instruction to rate each statement on a scale ranging from 1 (strong disagreement) to 5 (strong agreement). Examples of statements in the two domains are, “I am not afraid to voice my opinions even when they are in opposition of most people” (autonomy), and “Many daily activities often seem trivial and unimportant to me” (purpose in life). Some items are negatively phrased and need to be reversed when computing a sum score so that higher scores on all items indicate higher well-being. Scores are computed for each domain; a high score indicates a higher feeling of mastery. A sum score of both domains gives an indication of overall psychological well-being. Chronbach’s alpha in our dataset indicates rather low but acceptable internal consistency for group comparisons [32]: purpose in life α = 0.65 and autonomy α = 0.71.

##### Assistive Devices (ADs)

Questions regarding use and need of assistive devices (ADs) were adopted from the ENABLE-AGE Project, subsequently categorized according to ISO classifications [33]. This section covers ADs for communication, such as optical (three items) and hearing (three items), mobility devices indoors (six items) and outdoors (seven items), personal care (six items) and other ADs (seven items) such as stair lift and adjustable bed. In total, the participants answered 32 predefined questions regarding ADs with four response alternatives (available, in use, not available but would be necessary, not available, would not be necessary). If the participants expressed a need for or used an AD not listed, the project administrator used an open-ended question to register the responses.

#### 2.4.4. Objective Aspects of Home

Number of environmental barriers and magnitude of accessibility problems were captured with the Housing Enabler (HE) instrument which is administrated in three steps. The instrument is based on extensive research [34] and has proven to be valid and reliable [34,35]. Step 1 (the personal component checklist) of the HE concerns functional limitations (12 items; difficulty in interpreting information, visual impairment, blindness, loss of hearing, poor balance, incoordination, limitations of stamina, difficulties in moving head, reduced upper extremity function, reduced fine motor skill, loss of upper extremity skills, reduced spine and/or lower extremity function) and dependence on mobility devices (two items; dependence on walking devices, wheelchair). All items are dichotomously assessed as present/not present. The assessment results in a sum score of number of functional limitations (range 0–12) and dependence on mobility devices (range 0–2). This functional profile (12 + 2 items) can also be used as an objective aspect of health variable. Step 2 (the environmental component checklist) is based on observation of the actual environment in a detailed rating of environmental barriers (161 items). Each environmental barrier inside the home (*n* = 87), at entrances (*n* = 46) and in the immediate exterior environment (*n* = 28) is dichotomously assessed as present/not present. The ratings of the environmental component are based on national standards for housing design. Step 3 (the P-E fit analysis) involves calculating a total score that quantifies the magnitude of accessibility problems (MAP) in a particular case, and predicts the load caused by a particular combination of functional limitations and environmental barriers. The higher the score, the greater the accessibility problems are. The total score is always 0 if the individual has no functional limitations/dependence on mobility devices, regardless of the presence of environmental barriers. In addition, a rank order of the environmental barriers that cause the most accessibility problems (known as weighted environmental barriers), at individual or group level, can be calculated. Furthermore, participants were asked about how many years they had lived in the same home, how many rooms there were in their home, and how many people lived there.

#### 2.4.5. Perceived Aspects of Home

Perceived aspects of home were captured using a four-domain model, operationalized and empirically tested as described by Oswald *et al.* [36].

##### Domain 1: Housing Satisfaction

Housing satisfaction was assessed via the single question “Are you happy with the conditions of your home?”, adapted from the Housing Option for Older People (HOOP) Questionnaire (Sixsmith and Sixsmith, unpublished ENABLE AGE working paper). A five-point rating scale ranging from 1 (no, definitely not satisfied) to 5 (yes, definitively satisfied) was presented to the participants.

##### Domain 2: Usability

The Usability in My Home (UIMH) questionnaire was used to capture to what degree the physical environment supports the performance of daily activities in the home [37,38]. We used a short version containing 10 items divided in two subscales targeting activity aspects (4 items), for example, “In terms of how you normally manage your cooking/heating of food or preparation of snacks, to what extent is the home environment suitable designed in relation to this?”, and physical environmental aspects (6 items) of usability, for example, “How usable do you feel that your home environment is in general?”. The items are rated on a five-point scale ranging from 1 (not at all suitable/usable) to 5 (fully suitable/usable); higher scores mean higher usability. Chronbach’s alpha in our dataset indicates acceptable internal consistency [32] in both domains, that is, activity aspects, α = 0.72, and physical environmental aspects, α = 0.79.

##### Domain 3: Meaning of Home

The 28-item Meaning of Home (MOH) questionnaire was used to gain knowledge of the individual’s subjective meanings in relation to home. The MOH was developed to be used with older people and captures four aspects of the meaning of home: behavioral (6 items), for example, “doing everyday tasks”, physical (seven items) “feeling that home has become a burden”, cognitive/emotional (10 items) “feeling safe” and social (five items) “being excluded from social and community life”. Each item is rated on a scale with 11 response alternatives ranging from 0 (strongly disagree) to 10 (strongly agree). Higher scores indicate a stronger bonding/attachment to home [36]. In accordance with previous studies [8,9,36], Chronbach’s alpha in our dataset indicates rather low but acceptable internal consistency for group comparisons [32]: physical aspects, α = 0.53; behavioral aspects, α = 0.59; cognitive/emotional aspects, α = 0.62; and social aspects, α = 0.62.

##### Domain 4: Housing-Related Control Beliefs

Control beliefs in relation to home were addressed using the 24-item Housing-related Control Beliefs Questionnaire (HCB) [39]. The HCB captures three domains: internal control (8 items, sum-score), external control-powerful others (8 items, sum-score), and external control-chance (8 items, sum-score). Internal control denotes that housing-related outcomes are dependent on own behavior “Everything in my home will stay the way it is no one is going to tell me what to do”. External control means that an external power such as another person is responsible or that things happen by luck, chance, or fate. External control-powerful others is captured through statements like “In order to do anything interesting outside of my home I have to rely on others” whereas external control-chance is determined through statements like “Having a nice place is all luck. You cannot influence it; you just have to accept it”. The participants were asked to rate each statement on a five-point rating scale ranging from 1 (I do not at all agree) to 5 (I agree very much); higher scores indicate higher perceived control in the domain of internal control whereas higher scores in the domains of external control indicate lower perceived control. In accordance with previous studies [8,9,36], the domain of internal control will be excluded from further analysis due to low internal consistency (α = 0.38). Also similar to the previous studies, the two domains of external control reached rather low levels: powerful others, α = 0.54 and chance α = 0.56. Applying the same strategy as in studies based on data collected within the ENABLE-AGE Project [7,9] after combining the two dimensions of external control the 16-item scale reached an acceptable level [32] of internal consistency α = 0.69.

##### Housing Adaptations

Information on housing adaptations was gathered through five study-specific questions. The participants were asked whether they had knowledge about the housing adaptation grant provided by local municipalities (yes or no). Thereafter, with the same response alternatives, they were asked if there had been any adaptations made in the home. In cases where the participants answered yes, they were asked to provide information on the location of the adaptation as well as on how it had been financed. Moreover, the participants were asked if the adaptation had any positive or negative influences on ADL, using six response alternatives (it has become easier to perform my daily activities, I have been independent from help of others, I was able to remain living in the present dwelling, the changes had small/no effect, the situation has worsened, and other).

##### Neighborhood Attachment

Neighborhood attachment was captured through the single item “Are you rooted and feel a strong affinity to your residential area?”. The question has four response alternatives ranging from 1 (to a great extent) to 4 (not at all) [40].

All variables including the domains covered are presented in Table 1.

**Table 1:** Instruments and domains covered.

Instrument	Domain	Items, *n*	Literature Reference
Objective aspects of health			
Activities of daily life (ADL) Staircase	Personal ADL	5	[24]
	Instrumental ADL	4	
Perceived aspects of health			
Geriatric Depression Scale (GDS)	Mood Disturbance	10	[26]
	Motivation Disturbance	5	
Difficulty in ADL	Activity performed with/without difficulty^1^	2	[25]
Functional independence (PFI)	Perceived functional independence	1	-
Short FES-I	Fear of falling	7	[27,28]
Falls		4	-
Symptom list	Depression symptoms	5	[29]
	Tension symptoms	5	
	Gastrointestinal symptoms	8	
	Musculoskeletal symptoms	3	
	Metabolism symptoms	4	
	Heart-lung symptoms	3	
	Head symptoms	5	
SF-36, global health	Perceived global health	1	[30]
Physical mobility	Perceived physical mobility	1	-
Life Satisfaction	Life Satisfaction	1	-
Psychological wellbeing (PWQ)	Autonomy	10	[31]
	Purpose in life	9	
Objective environmental aspects			
Housing Enabler (HE)	Functional limitations/dependence on mobility devices ^2^	14	[34,35]
	Environmental barriers; Exterior surroundings	28	
	Environmental barriers; Entrance	46	
	Environmental barriers; Indoors	87	
Other aspects of objective housing	No. of rooms, no. of people, years of habitance	3	-
Assistive Devices/Technical Aids	Optical aids	3	[33]
	Hearing aids	3	
	Mobility devices, indoors	6	
	Mobility devices, outdoors	7	
	ADL devices	6	
	Other assistive devices	7	
Perceived environmental aspects			
Housing Option for Older People (HOOP)	Housing satisfaction^3^	1	
Usability In My Home (UIMH)	Activity	4	[37,38]
	Physical environmental aspects	6	
Meaning of Home (MOH)	Activity	6	[36]
	Physical	7	
	Cognitive/emotional	10	
	Social	5	
Housing Related Control Beliefs (HCB)	External control combined	16	[39]
Housing adaptations		5	-

^1^ Number of items used with each participant depends on the results of the objective assessment of ADL according to the ADL Staircase [24]. ^2 ^Used separately, the personal component of the Housing Enabler can also be used as a health variable. ^3 ^Sixsmith, A.J and Sixsmith, J.A, unpublished ENABLE AGE working paper.

### 2.5. Questions Regarding Reliability

In order to ensure the quality of the collected data the project administrator answered eight questions in order to evaluate the perceived reliability of the participant’s responses. This procedure was performed shortly after the home visit, without any contribution of the participant. It was noted if another person had been present during the interview, and if so, in what way the presence of this person might have influenced the responses given by the participants. The project administrator also registered the perceived communication ability of the participant (scored 0–10; higher = better) and her assessment of the reliability of the participant’s responses (very high reliability, high reliability, reliable, low reliability, very low reliability). Moreover, the status of the dwelling (neglected, normal, or well kept) and of the participant (neglected, average, well presented) as perceived by the project administrator were registered.

### 2.6. Data Collection

After project-specific training provided by experienced scientists with profound knowledge on the ENABLE-AGE data collection format, two project administrators (experienced registered occupational therapists) collected the data. To be able to administer the HE instrument, the project administrators completed a four-day training course and additional recommended practical training [41]. Data were collected at home visits over a 9-month period (5 October, 2010 to 21 June, 2011). Each home visit lasted 2.0–2.5 hours. In cases where it was not possible to complete the data collection during one home visit, a second appointment was made for completion of the data collection within 10 days.

### 2.7. Ethics

The Home and Health in the Third Age Study was conducted in accordance with the Helsinki Declaration and was approved by the Ethical Board in Lund (2010/431). Informed consent was obtained from all participants and anonymity was ensured. This was reinforced verbally as well as by means of written information at the start of the home visit. Participants were informed that they could withdraw from the study if and whenever they wished.

### 2.8. Data Quality Control

In order to monitor progress and data quality, meetings were arranged regularly with an experienced researcher (third author; M.H.) during the entire data collection period. A proof reading procedure was carried out to ensure the database accurately reflects the data collected. The proof reading included a sample of 40 randomly selected participants (>10%), and the acceptable error rate was set to not exceed 0.5%. Discrepancies found were noted on a log sheet and rectified in the database. The error rate was calculated to 0.18% (38 errors found among 40 individuals answering up to 520 variables), indicating that a 100% proof reading was not necessary. In addition, a validation of the data was performed by checking ranges, logical consistency and completeness. Missing or unclear data underwent a data cleaning process using data clarification forms. Changes applied to data in the database during the data cleaning process were noted on a log sheet. After completion of the data cleaning process the database was locked.

### 2.9. Data Analysis

For the empirical part of this paper, data collected with the following instruments were used: the ADL Staircase [24], SF-36 perceived global health [30], Symptoms list [29], Geriatric Depression Scale [26] and the HE instrument, Steps 1 and 2 [41]. Depending on the instrument scale properties, descriptive statistics were used and the findings reported with means and standard deviation (for continuous normally distributed data), medians and quartiles (for categorical data and data deviating from normal distribution), and frequencies and percentages (to describe group proportions). The Pearson Chi-Square test was used to test differences between sub-groups. The Mann-Whitney test was used to test for differences between medians, while the Student T-test was used to test differences between means. All tests were two-sided and *p*-values < 0.05 were considered statistically significant. All analyses were computed by means of the SPSS software version 20 (IBM Corporation, Armonk, NY, USA).

## 3. Results

### 3.1. Participants and Attrition Analysis

In the target sample of 673 men and women there were nine deaths. Consequently, 664 individuals (314 men, 350 women) were invited to participate. In all, 371 (55.9%) agreed to participate. At the start of the data collection, the mean age for participants was 68.4 years (SD = 0.9). Among the 293 individuals that declined to participate, 283 said they were unwilling without giving any reason, and 10 stated that their health was too poor to allow participation. A larger proportion of men than of women declined, 155 (52.9%) *vs.* 138 (47.1%) (*p* = 0.010). There was no age difference between participants and non-participants at time of recruitment: mean age 68.1 years (SD = 0.9) and 68.2 years (SD = 0.9), respectively (*p* = 0.324). Geographical area (urban or rural) did not differ between non-participants and participants. Among the non-participants, 224 (76.5%) lived in an urban area (*i.e.*, Malmö); among the participants the corresponding number was 287 (77.4%) (*p* = 0.783). For one participant, the HE assessment was not completed, but the remaining 370 participants completed all of the data collection.

### 3.2. Sample Characteristics

Description of the study sample according to sex, marital status, geographical area, type of housing, age and level of education is provided in Table 2. There were slightly more women than men participating in the study (*p* = 0.006). Out of the complete sample 64.2% were cohabiting (*p* ≤ 0.001) and 59.3% were living in multi-family buildings (*p* = 0.003). The majority were living in an urban environment (*p* ≤ 0.001). The distribution of participants living in rural and urban districts of our sample reflects the actual demographic distribution in the county of Skåne.

**Table 2:** Sample characteristics, N = 371.

Characteristic	N	%	*p*-value
*Sex*			
Women	212	57.1	
Men	159	42.9	0.006
*Marital Status*			
Married/cohabitating	238	64.2	
Unmarried	28	7.5	
Widow/widower	25	6.7	
Divorced/separated	67	18.1	
In a relationship	12	3.2	
Missing	1	0.3	<0.001
*Geographical Area*			
Rural	41	11.1	
Urban	330	88.9	<0.001
*Type of Housing*			
Apartment, owned in multi-family building	123	33.2	
Apartment, rented in multi-family building	97	26.1	0.003
One-family	151	40.7	
*Age in Years*			
67	158	42.6	
68	99	26.7	
69	87	23.5	
70	27	7.3	<0.001
*Education*			
Elementary school or less	139	37.5	
Secondary school	124	33.4	
One year more than secondary school or university degree	104	28.0	
Missing	4	1.1	0.080

### 3.3. Health and Home Aspects

As presented in Table 3, the vast majority of the participants rated their health as good or very good and there were no significant differences between sub-groups. Women reported more symptoms (*p* = 0.001) and had more functional limitations than men (*p* = 0.002). Approximately half of the participants (50.4%) had one or more functional limitation. For the men the most common functional limitation was “difficulty in bending or kneeling” whereas “difficulty in reaching with arms” was the most common among the women. Seventeen participants were reliant on a walking device and one participant used a wheelchair. Out of the total sample, 10% were dependent in one or more I-ADL whereas no participant was dependent in any P-ADL.

**Table 3:** Health variables and assistive devices for men and women in the study sample, N = 371.

Variable	Literature Reference	Men *n* = 159	Women *n* = 212	Total N = 371	*p*-value
Activities in daily life	[24]				
Independence in I-ADL, n (%)		137 (86.2)	197 (92.9)	334 (90)	0.031
Independence in P-ADL, n (%)		159 (100)	212 (100)	371 (100)	-
Perceived health Mn (Sd)	[30]	3.6 (0.95)	3.5 (1.05)	3.6 (1.01)	0.183
Symptoms (no.), Md (q1-q3)		5.0 (2.0-8.0)	6.5 (3.2-12.0)	6.0 (3.0-11.0)	0.001
Depressive symptoms Md (q1-q3)	[26]	1.0 (0.0- 1.0)	1.0 (0.0-2.0)	1.0 (0.0- 2.0)	0.066
Functional profile	[41]				
Functional limitations (n), Mn (Sd)		0.77 (1.07)	1.17 (1.47)	1.0 (1.33)	0.002
*Dependence on mobility devices*					
Reliance on walking aids, n (%)		4 (2.5)	13 (6.1)	17 (4.6)	0.099
Wheelchair user, n (%)		1 (0.6)	-	1 (0.3)	0.248

In every home environment investigated environmental barriers were identified. As presented in Table 4, environmental barriers were more common in multi-family than in one-family type of housing (*p* =< 0.001). There were no differences between the dwellings of men and women. The most prevalent environmental barriers were identified indoors in the kitchen and/or hygiene area (Table 5). In both multi-family and one-family housing “use requires hands” and “controls in high/low/inaccessible position” were identified as the most common environmental barriers. In the close exterior surroundings the most prevalent environmental barriers in the total sample were “irregular walking surface” (85.2%), “landscape furniture placed in the path of travel” (69.3%) and “narrow parking spaces” (67.1%).

**Table 4:** Number of environmental barriers in relation to type of housing and sex, N=371.

Environmental Barriers/Housing Section	Multi-Family Dwellings *n* = 220	One-Family Dwellings *n* = 151	*p*-value	Men *n* = 158	Women *n* = 212	*p*-value
Exterior surroundings, Mn (Sd)	11 (2.9)	7.5 (2.8)	<0.001	9.3 (3.4)	9.8 (3.1)	0.186
Entrances, Mn (Sd)	17.8 (6.7)	8.9 (3.2)	<0.001	13.6 (6.9)	14.6 (7.0)	0.235
Indoors, Mn (Sd)	43.0 (5.1)	48.7 (6.1)	<0.001	45.2 (6.4)	45.4 (6.1)	0.745
Total , Mn (Sd)	71.7 (9.9)	65.2 (8.4)	<0.001	68.2 (10.4)	69.7 (9.4)	0.135
Min-Max	44–95	45–86		0–91	45–95	

**Table 5:** The 20 most frequent environmental barriers at entrances and indoors, in different types of housing, N = 371.

Housing Section and Environmental Barrier ^1^	Multi-Family Dwellings *n* = 220	One-Family Dwellings *n* = 151	Total N = 371	*p*-value
Entrances	n (%)	n (%)	n (%)	
High thresholds and/or steps (more than 15 mm), (sitting-out place/balcony)	208 (94.5)	138 (91.4)	346	0.357
Kitchen, laundry room, utility kitchen				
No working surfaces with leg room	209 (95.0)	141 (96.4)	350 (94.3)	0.676
Turning motion of wrist required	217 (98.6)	149 (98.7)	366 (98.7)	0.524
Use requires hands	220 (100)	150 (99.3)	370 (99.7)	-
Use requires fingers (*i.e.*, isolated grip, e.g., pinch and lateral grip)	219 (99.5)	145 (96.0)	364 (98.1)	0.031
Controls in high/inaccessible position (more than 1.1 m above the floor)	220 (100)	150 (99.3)	370 (99.7)	-
Controls in low position (less than 80 cm above the floor)	220 (100)	150 (99.3)	370 (99.7)	-
Hygiene area				
Turning motion of wrist required	209 (95.0)	141 (93.4)	350 (94.3)	0.875
Use requires hands	219 (99.5)	149 (98.7)	368 (99.2)	0.410
Controls in high/inaccessible position (more than 1.1 m above the floor)	218 (99.1)	148 (98.0)	366 (98.7)	0.803
Wash-basin placed at a height for use only when standing	204 (92.7)	139 (92.1)	343 (92.5)	0.836
Toilet 47 cm or lower	199 (90.5)	143 (94.7)	342 (92.2)	0.046
Mirror placed at a height for use only when standing			360 (97.0)	0.701
Storage cupboards, towel hooks, *etc.* placed high/low	207 (94.1)	132 (87.4)	339 (91.4)	0.083
Inappropriate design of wardrobes/ clothes cupboards	194 (88.2)	142 (94.0)	336 (90.6)	0.058
Turning motion of wrist required	217 (98.6)	147 (97.4)	364 (98.1)	0.986
Use requires hands	220 (100)	149 (98.7)	369 (99.5)	0.225
Use requires fingers	216 (98.2)	149 (98.7)	365 (98.4)	0.346
Controls in high/inaccessible position (more than 1.1 m above the floor)	217 (98.6)	147 (97.4)	364 (98.1)	0.634
Controls in low position (less than 80 cm above the floor)	220 (100)	150 (99.3)	370 (99.7)	-

^1 ^No barriers in the immediate outdoor environment were found among the top 20 most frequent environmental barriers [41].

## 4. Discussion

According to the aim of this paper we present basic home and health characteristics of the Home and Health in the Third Age Study sample. The results show that environmental barriers are more common in multi-family than in one-family dwellings. Considering that a large number of people in the third age lives in multi-family housing [10] and will continue to do so, attention should be paid to removing environmental barriers in the already existing housing stock as well as when planning for new buildings. Especially since the current trend in Sweden and other western countries favors community-based health care and social services provided in ordinary housing before assisted living and institutional care [12].

Overall, it was beneficial to build the present study on the core methodology developed and tested within the internationally acknowledged ENABLE-AGE Project [23]. The main aim of the ENABLE-AGE was to explore the home environment as a determinant for autonomy, participation, and well-being in very old age within a follow-up perspective. For the present project we made use of the instruments, data collection and data quality assurance procedures that, based on our previous research (see e.g., 23), have been proven as the most efficient and valuable for studies with a specific focus on home and health in old age. To date, 50 original papers have been published based on the quantitative and qualitative data collected within the aforementioned project, and the present study was thus built on a strong knowledge base and methodological experiences gained during one decade of research.

As in line with previous findings [42], physical environmental barriers were identified in 100% of the homes assessed. This might be surprising, but since the HE environmental assessment [41] is very detailed and based on the present Swedish standards for housing design, virtually all housing units have some environmental barriers. Accordingly, when investigating the frequency of environmental barriers we found that among the 20 most common, four were identified as present in all of the 370 dwellings of the sample. Therefore, it is important to note that to understand the impact of the environmental barriers on aspects of health, in further studies based on the present sample analyses linking environmental barriers to the person’s functional limitations will be accomplished.

Studying a sample of people in the third age, the low prevalence of functional limitations was expected and in line with previous research [3]. However, earlier research has shown that the number of functional limitations and use of mobility devices will increase with advancing age [11]. As the number and profile of functional limitations and use of mobility devices are one component of accessibility [41], the magnitude of accessibility problems will also increase as people age [42]. As we have verified that the number of functional limitations is low among people in the third age, this study is as a starting point for future studies with the aim to identify turning points in home and health dynamics during the process of aging.

It should be noted that the Home and Health in the Third Age Study addresses key questions on the home and health interactions among individuals in an earlier phase of aging than those of previous studies with a similar focus and design (see e.g., [9,43]). An important goal in health promotion is to create home environments that support healthy aging [23]. Consequently, from a societal planning perspective it is of great importance to consider aspects of home and health from an earlier stage of aging and into very old age. To the best of our knowledge, no major studies exist that include detailed high-quality survey data not only on aspects of health but also on aspects of home among people in the third age. Under the canopy of a comprehensive research program labeled “Home and Health along the Process of Aging”, we are in a strong position to be able to deliver comparative studies, comparing this sample with other datasets with similar data on very old individuals [23]. Having access to a rich database will allow us to accomplish in-depth studies on home and health dynamics. Furthermore, we also have the possibility to compare this dataset with similar data on individuals aging with a chronic neurological disease [44] or a spinal cord injury [45] to shed new light on home and health dynamics along the process of aging. We will also be able to conduct longitudinal studies within the context of the SNAC/GÅS [21] utilizing data from previous and forthcoming data collection waves.

The fact that the project administrators assigned with the data collection task were responsible for contacting their respective participants presumably had a positive effect on the participation ratio. Still, the participation ratio was most likely somewhat negatively affected by the sometimes long waiting time between the invitation and the follow-up phone call. Furthermore, albeit resource intensive, face-to-face interviews administered at home visits have several advantages. In particular for participants with poorer health, continuity in the process of recruitment, fieldwork and individualized data collection at home visits are advantageous. Prior to the data collection, the project administrators obtained the necessary skills through training to use the instruments included in the survey provided by senior researchers with longstanding experiences of this kind of data collection. They were instructed to provide explicit information and give examples whenever a participant did not directly understand a question. That is, the project administrators made great efforts to tailor the data collection situation in an optimal way for each participant while still keeping up a high level of structure. Based on our extensive experiences from data collection with older people in similar projects, this procedure was used to make sure that the data collected is of the highest possible quality. Without any kind of influence on their responses as such, all the participants were given the same guidance to optimize their understanding of the question or item at hand.

Moreover, the home visit format was necessary for the administration of the objective assessment of the home environment by means of the HE instrument [41]. A noteworthy challenge for reliable administration of data on physical environmental features is the dynamics of outdoor environments. For example, since the data collection was carried out over a 9-month period in a Nordic country, the seasonal variation posed certain challenges. During winter the climate in southern Sweden is rather cold, sometimes with ice and snow. Naturally, this influenced the administration of the outdoor environmental assessment since the project administrator was not always in a position to rate specific items as present or absent due to weather conditions, resulting in some unintentional but unavoidable missing data. Furthermore, since the proportion of participants assessed as being influenced by another person during the interview was as low as 1.3% and only 1.1% were assessed as communicating with very low reliability, we conclude that the influence of these responses on the results are negligible.

Regarding the psychometric aspects of the instruments used, they all have documented validity and reliability based on studies including individuals in the fourth age (see e.g., 15, 23). Based on earlier observations [8,9,36], for the present study we concentrated our efforts regarding psychometric properties to the instruments that target perceived aspects of housing. In accordance with our studies on samples of people in the fourth age and a recent study by Oswald *et al.* [46], these instruments demonstrated low internal scale consistency also when used with people in the third age. As to the other instruments used in the present study, based on the data collected with people in the third age now at hand we are in a strong position to be able to contribute to further methodological refinement and development, aiming for the optimization of quantitative assessments of aspects of home and health in different phases of the aging process. Based on our earlier studies, valid data treatment and analysis strategies have been established [9,36], and we are thus well equipped to deliver forthcoming studies based on the data collected.

## 5. Conclusions

The present study will generate a better understanding of home and health dynamics among people in the third age. Future results have the potential to contribute to and facilitate societal planning, particularly in terms of housing provision but also regarding home care and social services for senior citizens.

## Abbreviations

ADL: Activities of Daily Living
I-ADL: Instrumental Activities of Daily Living
P-ADL: Personal Activities of Daily Living
HE: Housing Enabler
MAP: Magnitude of Accessibility Problems
ENABLE-AGE: Enabling Autonomy, Participation, and Well-Being in Old Age
MOH: Meaning of Home
HCB: Housing Related Control Beliefs
UIMH: Usability in My Home
GDS: Geriatric Depression Scale
PWQ: Psychological wellbeing
HOOP: Housing Options for Older People
SNAC: Swedish National Study on Aging and Care
GÅS: Gott Åldrande i Skåne
AD: Assistive Device
FES-I: Falls Efficacy Scale-International
FOF: Fear of Falling
